# Phytochemical Profiling of the Leaf Extract of *Ximenia americana* var. *caffra* and Its Antioxidant, Antibacterial, and Antiaging Activities *In Vitro* and in *Caenorhabditis elegans*: A Cosmeceutical and Dermatological Approach

**DOI:** 10.1155/2022/3486257

**Published:** 2022-03-28

**Authors:** Widad Ben Bakrim, Agustina Dwi Retno Nurcahyanti, Malak Dmirieh, Ismail Mahdi, Abdelbaset M. Elgamal, Mohamed A. El Raey, Michael Wink, Mansour Sobeh

**Affiliations:** ^1^AgroBioSciences, Mohammed VI Polytechnic University, Lot 660, Hay Moulay Rachid, Ben Guerir 43150, Morocco; ^2^African Sustainable Agriculture Research Institute (ASARI), Mohammed VI Polytechnic University (UM6P), Laayoune, Morocco; ^3^Department of Pharmacy, School of Medicine and Health Sciences, Atma Jaya Catholic University of Indonesia, Pluit Raya 2, 14440 Jakarta, Indonesia; ^4^Institute of Pharmacy and Molecular Biotechnology, Heidelberg University, Im Neuenheimer Feld 364, 69120 Heidelberg, Germany; ^5^Department of Chemistry of Microbial and Natural Products, National Research Centre, Dokki, Cairo 12622, Egypt; ^6^Department of Phytochemistry and Plant Systematics, National Research Centre, Dokki, Cairo 12622, Egypt

## Abstract

We previously annotated the phytochemical constituents of a root extract from *Ximenia americana* var. *caffra* and highlighted its hepatoprotective and hypoglycemic properties. We here extended our study on the leaf extract and identified its phytoconstituents using HPLC-PDA-ESI-MS/MS. In addition, we explored its antioxidant, antibacterial, and antiaging activities *in vitro* and in an animal model, *Caenorhabditis elegans*. Results from HPLC-PDA-ESI-MS/MS confirmed that the leaves contain 23 secondary metabolites consisting of condensed tannins, flavonol glycosides, flavone glycosides, and flavonol diglycosides. The leaf extract demonstrated significant antioxidant activity *in vitro* with IC_50_ value of 5 *μ*g/mL in the DPPH assay and 18.32 *μ*g/mL in the FRAP assay. It also inhibited four enzymes (collagenase, elastase, hyaluronidase, and tyrosinase) crucially involved in skin remodeling and aging processes with comparable activities to reference drugs along with four pure secondary metabolites identified from the extract. In accordance with the *in vitro* result, *in vivo* tests using two transgenic strains of *C. elegans* demonstrated its ability to reverse oxidative stress. Evidence included an increased survival rate in nematodes treated with the prooxidant juglone to 68.9% compared to the 24.8% in untreated worms and a reduced accumulation of intracellular reactive oxygen species (ROS) in a dose-dependent manner to 77.8%. The leaf extract also reduced levels of the expression of HSP 16.2 in a dose-dependent manner to 86.4%. Nuclear localization of the transcription factor DAF-16 was up to 10 times higher in worms treated with the leaf extract than in the untreated worms. The extract also inhibited the biofilm formation of *Pseudomonas aeruginosa* (a pathogen in skin infections) and reduced the swimming and swarming mobilities in a dose-dependent fashion. In conclusion, leaves of *X. americana* are a promising candidate for preventing oxidative stress-induced conditions, including skin aging.

## 1. Introduction

The aging population is increasing fast as people worldwide are living longer. It is estimated to reach one-sixth of the global population by 2030. Many human organs change structure and function with increasing ages, including the skin. Age-related physiological changes in skin structure, integrity, and function can cause vulnerability to many dermatology problems such as pruritus, dermatitis, and infections [1]. Skin aging is closely associated with degradation and disorganization of collagen in the dermis and at the dermal-epidermal junction, causing deterioration of epidermal cell-cell junctions. Consequently, aging skin experiences a decline in the skin barrier function, changing the skin microenvironment and cutaneous immunity system, and therefore, the skin becomes susceptible to bacterial infections [2].

Aging is a natural process triggered by the presence of intracellular and extracellular reactive oxygen species (ROS), including skin aging, which is characterized by wrinkles and changes on pigmentation [3]. Natural antioxidant can prevent several conditions related to aging [4], including dermal aging. The mechanisms include the inhibition of key enzymes of aging, namely, collagenase, elastase, hyaluronidase, and tyrosinase [4]. Collagenase and elastase are fundamental enzymes that degrade major components of the extracellular matrix, collagen, and elastin and thus accelerate wrinkle formation and its featuring skin aging [3]. Hyaluronidase is a proteolytic enzyme responsible for the degradation of hyaluronan in the extracellular matrix. Likely, loss of hyaluronan causes wrinkles and sagging in the skin [3]. Tyrosinase is an enzyme that converts tyrosine to melanin [5], and thus, inhibition of tyrosinase activity plays an important role in skin-lightening process [6].

ROS as well as nonradical derivatives of oxygen and nitrogen are formed as by-product from aerobic metabolic metabolism in mitochondria [7]. ROS can be also generated as a cellular response to xenobiotics, cytokines, microbial attack, radiation, and environmental pollution [8]. An overproduction of ROS can cause oxidative stress. The latter represents the imbalance of radicals or oxidants, beyond the capacity of the endogenous antioxidant enzymes and effective antioxidant response, resulting in macromolecular and tissue damage [9]. Especially important are mutations caused by ROS in that the DNA base guanosine is converted to 8-oxoguansosine, which pairs with adenosine instead of cytosine. Causing intracellular damage and mutations, oxidative stress is directly implicated in the mechanism and progression of several diseases such as diabetes, metabolic syndrome, obesity, atherosclerosis, cancer, and neurodegeneration and aging [9].

Natural phytochemicals (flavonoids, tannins, carotenoids, vitamins C and E, mustard oils, and allicin) from vegetables, fruits, and herbs are known for their antioxidant capacity and anti-inflammatory and antibacterial properties, making them excellent candidates to improve aging skin and develop into low-cost, high-efficacy, and tolerance dermo-cosmetic products [10, 11].

*Ximenia* constitutes one of 25 genera of the family Oleaceae. The large sour plum, *Ximenia americana* var. *caffra*, is a deciduous tree, native to Africa, and is cultivated from Tanzania in East Africa to South Africa, crossing Namibia and Botswana. The plant is also distributed in other tropical regions such as India, South East Asia, Australia, New Zealand, Pacific Islands, West Indies, and Central and South America [12]. *X. americana* has been prescribed as an herb in traditional medicine for ages [13].

Several pharmacological activities have been reported from the different parts of the plant. These include antibacterial, antifungal, antioxidant, antiproliferation, anti-inflammatory, antidiabetic, and hepatoprotective properties. For instance, we previously described pronounced hypoglycemic and hepatoprotective effects of a proanthocyanidin-rich extract from the roots from plants originating from Tanzania. These activities were attributed to the presence of 20 secondary metabolites, among them are a series of catechin, epi-catechin, (epi)-catechin-(epi)-catechin-(epi)-catechin, and proanthocyanidin dimer monogallate derivatives [13]. The leaf extract from plants of northeast of Namibia exhibited anti-inflammatory, antiproliferation, and antioxidant activities along with the confirmation of 10 polyphenols including catechin, gallic acid, kaempferol, and quercetin and their derivatives [14]. Potential phytochemicals from an ethanol extract of leaves from plants from Mali have been also elucidated; they consisted of a cyanogenic glycoside, sambunigrin, gallic acid, glucogalline, 1,6-digalloyl glucopyranose, quercetin, quercitrin, avicularin, quercetin-3-*O*-*β*-xylopyranoside, quercetin-3-*O*-(6^″^-galloyl)-*β*-glucopyranoside, and kaempferol-3-*O*-(6^″^-galloyl)-*β*-glucopyranoside. Many of them contributed to the antioxidant activity and xanthine oxidase and 15-lipoxygenase inhibitory activities [15].

In this study, we extended our investigation on the chemical composition of the leaf extract from Tanzania using HPLC-PDA-ESI-MS/MS, as no study from east African plants has been found and evaluated its antioxidant and antiaging effects *in vitro* using DPPH and FRAP assays and the inhibition of four enzymes involved in skin aging, namely, collagenase, elastase, hyaluronidase, and tyrosinase by quinic acid, rutin, catechin, and epicatechin, compounds identified from the extract. Furthermore, we investigated the biofilm inhibition activities of the extract against the common encapsulated gram-negative bacteria, *Pseudomonas aeruginosa*, a common pathogen in skin infections. *Caenorhabditis elegans* was employed as an animal model for studying the antioxidant properties of the leaf extract.

## 2. Material and Methods

### 2.1. Plant Material and Extraction

Plant leaves were gathered from the Lupaga site in Shinyanga, Tanzania. DNA barcoding techniques amplifying the *rbc*L fragment were used to identify the plant. Upon the identification, plant specimens were deposited at the Institute of Pharmacy and Molecular Biotechnology, Heidelberg University, under accession number P7344 [16]. Dried leaves (100 g) were powdered before being subjected to methanol for extraction at room temperature for 3 days. The extract was filtered and then evaporated under optimum vacuum condition, and the obtained residue was freeze-dried to -70°C to yield fine dried powder (19 g).

### 2.2. HPLC-PDA-ESI-MS/MS

The composition of the leaf constituents was analyzed using a Thermo Finnigan system (Thermo Electron Corporation, USA) tandem with LCQ-Duo ion trap mass spectrometer with an ESI source (ThermoQuest) as described in our previous study [17]. Separation of constituents was attained using Zorbax Eclipse XDB-C18 (4.6 × 150 mm, 3.5 *μ*m, Agilent, USA). Gradient eluent is composed of water and acetonitrile with 0.1% formic acid in each eluent, from 5 to 30% acetonitrile in 60 min, and flow rate 1 mL/min with a split ratio of 1 : 1 to enter the ESI interface. Xcalibur software (Xcaliburۛ 2.0.7, Thermo Scientific) was used to command the machine [13].

### 2.3. Total Phenolic and Antioxidant Activity *In Vitro*

Total phenolic content was estimated using the Folin–Ciocalteu method as described in a previous study [18], while antioxidant activity was determined using two colorimetric methods, DPPH assay [19] and FRAP assay [20]. The analysis was performed as in the previous study [18].

### 2.4. Enzymatic Activities

#### 2.4.1. Collagenase Inhibition

The assay was performed according to a previous study [21]. Collagenase was obtained from *Clostridium histolyticum* (ChC—EC.3.4.23.3), and the assay was performed in 50 mM Tricine buffer consisting of 400 mM NaCl and 10 mM CaCl_2_ (pH 7.5). Collagenase was dissolved in the buffer to achieve an enzyme activity concentration of 0.8 unit/mL, and the synthetic substrate *N*-[3-(2-furyl) acryloyl]-Leu-Gly-Pro-Ala (FALGPA) was dissolved in the buffer to make up the final concentration of 2 mM. Serial diluted samples were incubated with the enzyme for 15 min. After that, the substrate was added to each serial solution. Absorbance was measured using a microplate reader (Costar, 96) at 490 nm. Each measurement was made in triplicate. Percentage of collagenase inhibition (%) was calculated according to the formula: inhibition (%) = [(*A*_control_ − *A*_sample_/*A*_control_) × 100].

*A*_sample_: absorbance value of the collagenase activity from samples or positive control (quercetin)

*A*_control_: absorbance value of the collagenase activity from negative control (solution without samples)

#### 2.4.2. Tyrosinase Inhibition

This assay was performed using mushroom tyrosinase and L-DOPA as substrates, according to a previous study [21] with slight modifications. The reaction mixture consisted of 20 *μ*L of mushroom tyrosinase (2500 U mL^−1^), 20 *μ*L of samples, 20 *μ*L of 5 mM L-DOPA, and 100 *μ*L of phosphate buffer (0.05 M, pH 6.5). After adding L-DOPA, the reaction mixture was monitored at 475 nm for dopachrome formation using a microplate reader (Costar, 96). Each measurement was made in triplicate. The percentage of tyrosinase inhibition (%) was calculated according to the formula: inhibition (%) = [(*A*_control_ − *A*_sample_/*A*_control_) × 100].

*A*_sample_: absorbance value of the tyrosinase activity from samples or positive control (kojic acid)

*A*_control_: absorbance value of the tyrosinase activity from negative control (solution without samples)

#### 2.4.3. Elastase Inhibition

This assay was performed according to a previous study [21]. Porcine pancreatic elastase was used in this assay at stock concentration of 3.33 mg/mL in sterile water. N-Succinyl-Ala-Ala-Ala-*p*-nitroanilide (AAAPVN) was used as a substrate and dissolved in buffer to make up 1.6 mM solution. A serial dilution of the extract with amount of 50 *μ*L each was incubated with the enzyme for 15 min, and the substrate was added subsequently and makes up the final reaction mixture of 250 *μ*L. Absorbance value was measured at 400 nm using a microplate reader. Each measurement was made in triplicate. The percentage of elastase inhibition (%) was calculated according to the formula: inhibition (%) = [(*A*_control_ − *A*_sample_/*A*_control_) × 100].

*A*_sample_: absorbance value of the elastase activity from samples or positive control (kojic acid)

*A*_control_: absorbance value of the elastase activity from negative control (solution without samples)

#### 2.4.4. Hyaluronidase Inhibition

The hyaluronidase inhibition assay was performed using hyaluronidase (1.5 mg/mL) and the substrate hyaluronic acid (1 mg/ml in 0.1 M acetate buffer; pH 3.5), according to a previous study [21]. The reaction mixture was prepared consisting of 25 *μ*L of CaCl_2_ (12.5 mM), 12.5 *μ*L of each test sample (serial dilution), hyaluronidase (1.5 mg/mL), and 100 *μ*L substrate hyaluronic acid. Afterwards, reaction mixture was placed in a water bath at 100°C for 3 min, and subsequently, 25 *μ*L of 0.8 M KBO_2_ was added. After cooling to the room temperature, 800 *μ*L DMAB (4 g DMAB in 40 mL acetic acid and 5 mL 10 N HCl) was added to the mixture following the incubation for 20 min. The absorbance was measured at 600 nm. Each measurement was made in triplicate. The percentage of hyaluronidase inhibition (%) was calculated according to the formula: inhibition (%) = [(*A*_control_ − *A*_sample_/*A*_control_) × 100].

*A*_sample_: absorbance value of the hyaluronidase activity from samples or positive control (quercetin)

*A*_control_: absorbance value of the hyaluronidase activity from negative control (solution without samples)

### 2.5. Antioxidant Activities *In Vivo*

#### 2.5.1. *Caenorhabditis elegans* Strains and Culture

*C. elegans* grows optimally at 20°C in NGM medium supplemented with living *E. coli* OP50. Age-synchronized animals were used for each experiment. EGCG from green tea served as a positive control for an antioxidant natural product. The synchronization was attained by treating gravid adult nematodes with sodium hypochlorite to hatch the eggs in M9 buffer. The growing larvae were transferred to S-media containing bacteria (OD_600_ = 1.0) (T 2006). Wild type (N2), TJ375 [P*hsp-16.2: GFP(gpls1)*], and TJ356 were obtained from the *Caenorhabditis* Genetic Center (CGC), University of Minnesota, USA, and they were used in the current study.

### 2.6. Survival Assay

This assay employed wild-type worms from an early larval stage (L1) as developed in other studies [22]. Extracts at different concentrations from 25 to 100 *μ*g extracts/mL were administered to L1 worms growing in S-medium at 20°C for 48 h. The control group received no extract. Oxidative stress was induced by adding the naphthoquinone juglone from *Juglans regia* at the final concentration of 80 *μ*M. After 24 h under stress conditions, living worms were counted and the results were presented as the percentage of living worms.

### 2.7. Inhibition of Intracellular ROS Production

Like in the survival assay, L1 wild-type worms were maintained in S-medium at 20°C and three different concentrations of the extract were applied for 48 h. Afterwards, the worms from each treatment were transferred to M9 buffer containing 20 *μ*M H2DCF-DA for further incubation at 20°C for 30 min before being observed under a fluorescence microscope (Keyence, BZ-9000, Osaka, Japan). Fluorescence intensity was quantified using ImageJ 1.48 software (National Institutes of Health, Bethesda, MD) [23].

### 2.8. Expression of Hsp-16.2: GFP Reporter

This assay employed the TJ375 transgenic strain with GFP fusion to heat shock protein (*hsp*) as an oxidative stress reporter. Early larval stage (L1) worms were maintained at S-medium at 20°C as developed in other studies [22]. After administration of three different concentrations on separated culture plates for 48 h, worms were subjected to stress oxidation using 20 *μ*M juglone for 24 h. Fluorescence intensity was observed using a fluorescence microscope and quantified using ImageJ 1.48 software.

### 2.9. Identification of Subcellular DAF-16: GFP Localization

This assay employed the TJ356 transgenic strain with GFP fusion to the DAF-16 transcription factor to observe the modulation of the extract in the regulation of stress response (P et al. 2016). Early larval stage (L1) worms were maintained at S-medium at 20°C for 24 h. After administration of three different concentrations for 24 h, localization of the DAF-16 transcription factor was observed using Keyence, BZ-9000, Osaka, Japan. DAF-16 localization was categorized as cytosolic, intermediate, and nuclear.

### 2.10. Antibacterial Activity

The antibacterial activities were evaluated using the broth microdilution assay using a 96-well microtiter microplate. The extract was two-fold serially diluted in MH broth (100, 50, 25, 12.5, 6.25, 3.125, and 1.562 mg/mL), filtered using 0.22 *μ*m sterile syringe filters, and transferred to the microplate's wells in quadruplicate (200 *μ*L per well). Next, 2 *μ*L of a fresh overnight bacterial culture of *P. aeruginosa* adjusted to OD_600 nm_ = 1 using MH broth was inoculated into each well of the microplate and incubated at 37°C under shaking at 150 rpm for 18 h. The minimum inhibitory concentration (MIC) was defined as the lowest concentration that inhibits visible microbial growth [24].

### 2.11. Bacterial Biofilm Inhibition Assay

The antibiofilm potential of the extract was assessed using a colorimetric assay [24, 25]. Firstly, the extract at the doses of 3.13 mg/mL (1/8 MIC) and 6.25 mg/mL (1/4 MIC) was prepared in Mueller Hinton (MH) broth and then filtered using 0.22 *μ*m syringe filters. Filtered extracts were transferred, in quadruplicate, into a 96-well microtiter microplate. Next, 2 *μ*L of a fresh overnight bacterial culture of *Pseudomonas aeruginosa* adjusted to OD_600 nm_ = 1 using MH broth was inoculated into each well of the microplate and incubated at 37°C under shaking at 150 rpm. Media without bacterial inoculation were used as negative controls. After 18 h of incubation, the culture suspensions were discarded, and the wells were washed four times with a phosphate-buffered saline solution to get rid of planktonic bacteria. To reveal the biofilm production, each well was stained with a 1% crystal violet solution and incubated for 15 min at room temperature. A second vigorous washing with distilled water was performed to remove excess dye. The produced biofilm was solubilized using 95% ethanol, and the absorbance of dye in ethanol was measured at OD_600 nm_ using a multimode plate reader. The attached biofilms to wells' surfaces were microscopically observed before and after solubilization by ethanol.

### 2.12. Swimming and Swarming Inhibition Tests

The extracts were studied for their effect on the swimming and swarming motility of *P. aeruginosa* on solid media. The swimming (1% tryptone, 0.5% sodium chloride, and 0.3% agar) and swarming (semisolid LB medium, 0.6%) media were aseptically supplemented with the doses of 3.13 mg/mL (1/8 MIC) and 6.25 mg/mL (1/4 MIC) of the extract. Afterwards, 7 *μ*L of a fresh overnight culture of *P. aeruginosa* (OD_600 nm_ = 1) was deposited at the center of each plate and incubated at 37°C for 24 h. The swimming and swarming zone diameters were measured in cm [24].

### 2.13. Statistical Analysis

Inhibitory concentration 50 (IC_50_) was determined by extrapolating the dose-response curve using GraphPad Prism software. IC_50_ value represents the capacity of the extract (substance) to inhibit 50% of radical oxidant- and enzyme-associated aging. Statistical analysis was performed using the Statistical Package of the Social Sciences (SPSS) version 23 (IBM SPSS software, USA) from at least three triplicate experimental data. The data were expressed as a mean ± SEM. Significant difference was analyzed using one-way analysis of variance (ANOVA) followed by the Duncan analysis. The presence of significant differences in treatment was at 95% confidence interval.

## 3. Results and Discussion

**Figure 1:**
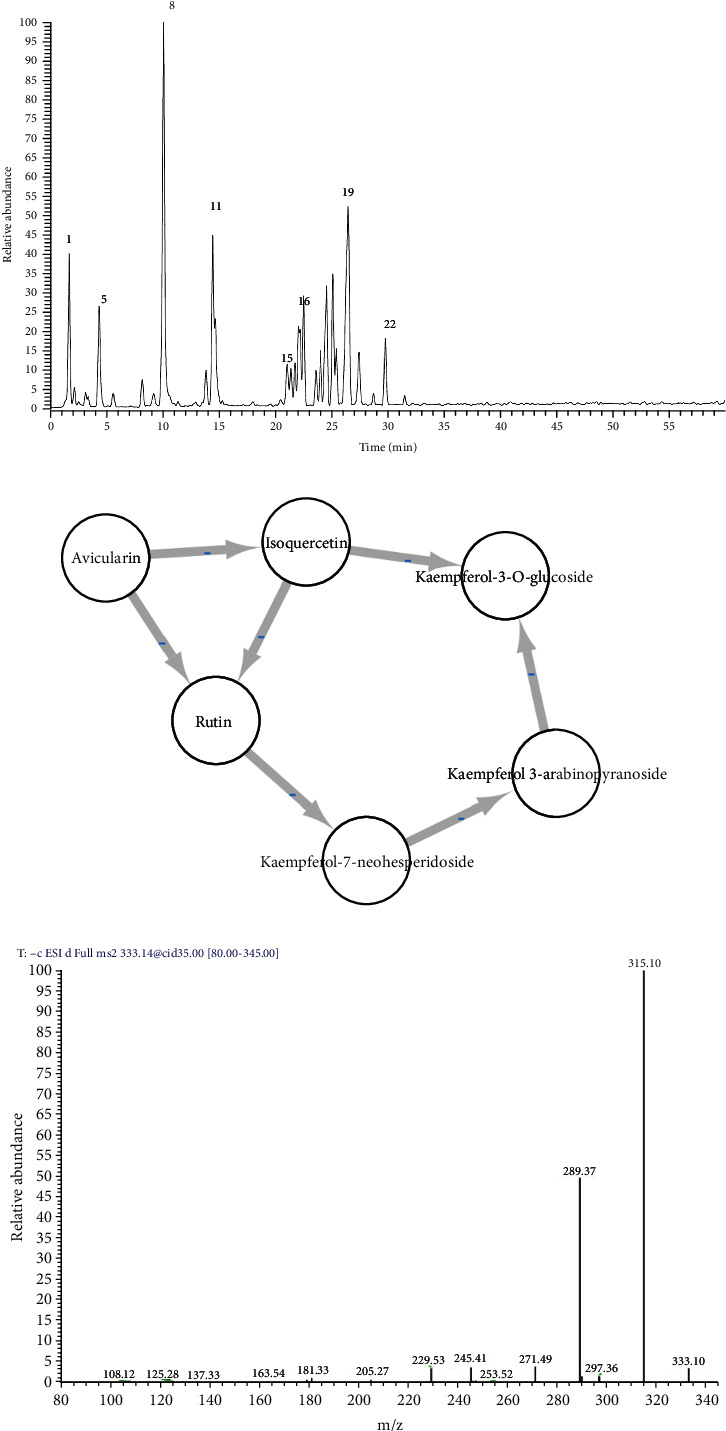
(a) LC-MS profile of *X. americana* var. *caffra* leaves. (b) Molecular network cluster of flavonoids. (c) MS/MS of catechin carboxylic acid (compound 5). Numbers of peaks refer to numbers in Table 1.

HPLC-PDA-ESI-MS/MS analysis of the leaf extract revealed 23 secondary metabolites representing several classes of quercetin-related compounds, kaempferol-related compounds, procyanidin B1, epicatechin, and catechin (Table 1 and Figure 1(a)). The compounds were tentatively identified according to their MS and MS/MS spectral data and retention times. The MS file was also utilized for generating a molecular network for a precise identification (Figure 1(b)). The flavonoid cluster furnished three quercetin-related compounds, namely, rutin, isoquercetin, and avicularin, and three kaempferol-related compounds including kaempferol 3-*O*-glucoside, kaempferol 3-neohesperidoside, and kaempferol 3-*O*-arabinoside (Figure 1(b)). Noteworthy, a signal at 4.38 min displayed an [M-H]^−^ at *m*/*z* 333 and four daughter ions at 315 [M-H-18]^−^, 289 [M-H-44]^−^, 245, and 205; it was tentatively identified as catechin carboxylic acid (Figure 1(c)). Some compounds share similar structure as found in the root extract, mainly the class of condensed tannin, such as flavan-3-ol (further referred to as catechin and epi-catechin), (*epi*)-catechin-(*epi*)-catechin-(*epi*)-catechin, and procyanidin dimer monogallate) [13]. The root extract did not reveal procyanidin B1, rutin, isoquercetin, avicularin, kaempferol 3-*O*-glucoside, kaempferol 3-neohesperidoside, and kaempferol 3-*O*-arabinoside, and here, we confirm that they are found only in the leaves.

### 3.1. Antioxidant Activities *In Vitro*

The extract revealed pronounced antioxidant activities in DPPH and FRAP assays (Table 2). Similar activities were also reported of the leaf extract from Namibia and the root extract from Tanzania [13, 14]. These activities can be attributed to the existence of high amount of phenolics of 451 mg gallic acid equivalent/g extract as determined by the Folin–Ciocalteu assay.

The presence of procyanidins remarkably contributes to the activity as demonstrated in many other antioxidant plants including *Schotia brachypetala* Sond. [23], grape seed (*Vitis vinifera* L.) [26], and cocoa (*Theobroma cacao*) [27]. Quercetin-related compounds (rutin, isoquercetin, and avicularin), found in the leaf extract, exhibit exceptional antioxidant activity [28]. In addition to quercetin, also flavonoid glycosides, such as those found in the leaf of *X. a.* var. *caffra*: kaempferol 3-*O*-glucoside, kaempferol 3-neohesperidoside, and kaempferol 3-*O*-arabinoside (Figure 1(b)), are responsible for important antioxidant activity [21, 29]. Many of flavonoid glycosides (which are the storage form in plant vacuoles) demonstrate higher solubility in water than their parent flavonoid as a consequence of hydrophilic property of the sugar moieties thus affecting the antioxidant capacity [30, 31]. For instance, the glycoside derivative of luteolin, orientin, demonstrates higher antioxidant activity than luteolin due to sugar substitution that decreases negative charge on one of the oxygen atoms, resulting in a higher activity of orientin [32]. *Hypericum perforatum* L., rich in rutin, hyperoside, isoquercitrin, avicularin, quercitrin, and quercetin, has demonstrated profound antioxidant activity including free radical scavenging activity, metal-chelation activity, and reactive oxygen quenching activity [33]. The presence of rutin, isoquercetin, and avicularin in *Malus domestica* leaves is responsible for their antioxidant activity [34].

### 3.2. Enzymatic Activities

In the current study, the leaf methanol extract of *X. a. caffra* was examined against four key enzymes of dermal aging (Table 3). The extract demonstrated elastase inhibitory and hyaluronidase activity comparable to the reference kojic acid (Table 3). We also explored the antiaging activities of four compounds from the extract and their furnished considerable activities (Table 3).

Plants rich in antioxidant compounds such as catechin, kaempferol, and quercetin and their related compounds revealed antiaging potential via inhibition activity against the mentioned aging key enzymes [4, 35]. Catechin, such as found in green tea, exhibits protective effect against skin stress-induced photoaging [36]. A plant extract, rich in rutin, hyperoside, isoquercitrin, avicularin, quercitrin, and quercetin, such as those from the genus *Hypericum*, has demonstrated profound antioxidant, antiaging, and antityrosinase activity [37]. The leaf methanol extract of *X. a. caffra* is rich in antioxidant compounds (Table 1); they can act synergistically to inhibit the activity of skin aging enzymes and thus potentially delay the skin aging process.

### 3.3. Antioxidant Activities *In Vivo*

**Figure 2:**
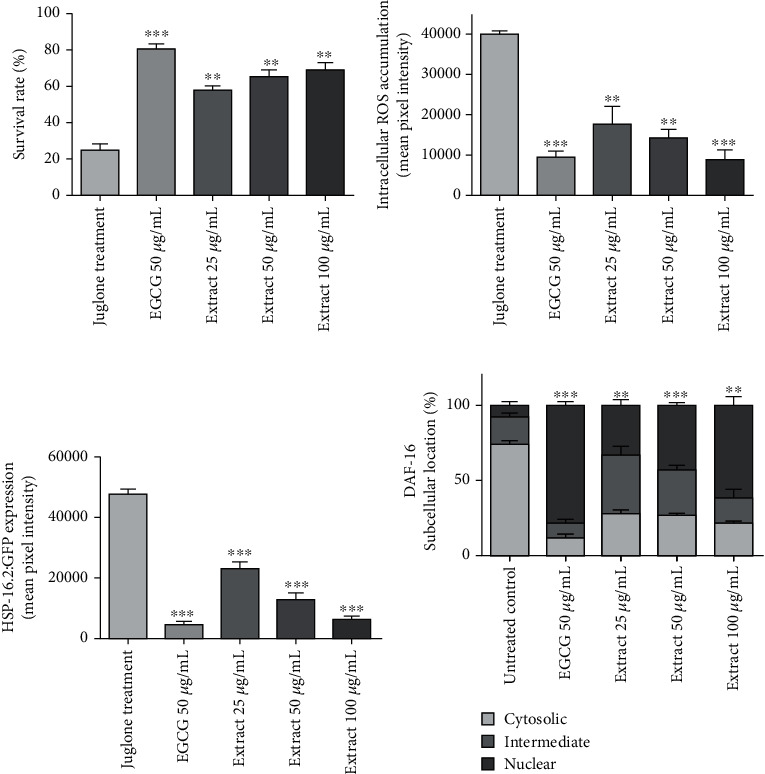
Treatments of *C. elegans* with the leaf extract from *X. americana*: (a) survival rate after challenge with juglone; (b) intracellular ROS concentrations; (c) HSP-16.2:GFP expression; (d) DAF-16 subcellular translocation. Results were expressed as mean (*n* = 3). Significant differences relate to the control group at ^∗∗^*p* < 0.01 and ^∗∗∗^*p* < 0.001 by one-way ANOVA and Tukey's *post hoc* test.

The *X. a. caffra* leaf extract was able to enhance the survival of nematodes under stress conditions induced by juglone. Increased concentration of the leaf extract significantly improved the number of surviving worms in a dose-dependent fashion (Figure 2(a)). Other experiments supported the antioxidant activities, one of them being the decreased amount of intracellular ROS production after treatment with the leaf extract in a dose-dependent manner, ranging from 55.87 to 77.75% of ROS reduction (Figure 2(b)). Quercetin and its related compounds have shown profound *in vivo* antioxidant activity, including the activity to regulate glutathione synthesis in animal and cell models [38, 39]. In addition, quercetin and rutin have also shown strong antioxidant enzymatic activity using a rat's brain homogenate [40], upregulating the expression of antioxidant enzymes heme-oxygenase- (HO-) 1 and superoxide-dismutase- (SOD-) 1 in primary human osteoblasts exposed to stress condition from cigarette smoke [41]. Quercetin can improve the antioxidant defense system by upregulating superoxide dismutase and catalase and reducing the level of malondialdehyde [42]. The presence of procyanidin B1, rutin, isoquercetin, avicularin, kaempferol 3-*O*-glucoside, kaempferol 3-neohesperidoside, and kaempferol 3-*O*-arabinoside only in the leaf extract may synergistically activate the intracellular antioxidant defense mechanism [43], thus reducing the production of ROS, preventing cell damage, and prolonging worm survival [23].

Heat shock proteins (Hsps) are a large family member of molecular chaperons that are ubiquitous proteins responsible for correcting protein refolding [44]. Hsps are upregulated after stress stimuli, such as xenobiotics, hyperthermia, and hypoxia [44]. Due to the biological role, Hsps have become a prominent biomarker for stress and thus has been developed for model organisms for undertaking experimental stress conditions in the laboratory, for instance, *C. elegans* [45]. Our study used transgenic *C. elegans* TJ375 in which small hsp and hsp-16.2p are coupled with the green fluorescence protein (GFP) reporter to detect the expression of hsp when exposed to stress conditions.  Figure 2(c) demonstrates that increasing concentrations of the extract can significantly reduce hsp-16.2 expression in juglone-treated worms, reported by a lower intensity of GFP fluorescence.

Another transgenic strain used in the current study was TJ365, in which the DAF-16 gene is coupled to the GFP protein. About 8 different genes are responsible and involved in dauer arrest and longevity. One of them is the transcription factor DAF-16/FOXO that modulates signals responsible for aging and longevity when migrating from the cytoplasm to the nucleus. Localization of DAF-16 into the nucleus indicates the response to stress condition and capacity to prevent damage and arrest cell and increased endurance and survival under stress condition [46]. Our current study has shown a 4 to 10 times higher nuclear DAF-16 localization in worms treated with the leaf extract in a dose-dependent manner, as compared to untreated worms (Figure 2(d)).

*Cassia abbreviata*, rich in epicatechin and other tannins, has demonstrated a modulation toward stress resistance in *C. elegans* [47]. The flavonoid kaempferol demonstrates translocation of the FOXO transcription factor DAF-16 from the cytosol to the nucleus in *C. elegans*, indicating that the flavonoid modulates aging and longevity signaling cascade [48]. Similar findings on increased stress resistance on *C. elegans* have also been shown by rutin via distinct pathways, including redox-sensitive signaling pathways [49]. Quercetin-related compounds have also demonstrated capacity to increase stress resistance and lifespan on *C. elegans* via the insulin-like signaling pathway and p38-mitogen-activated protein kinase pathway [50].

### 3.4. Effects of the Extract on Biofilm Inhibition and Swimming and Swarming Mobilities of *P. aeruginosa*

**Figure 3:**
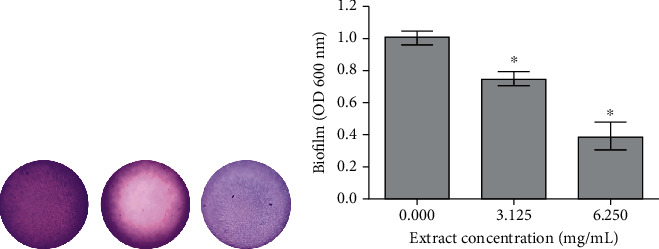
(a) Effect of the leaf extract from *X. americana* on the biofilm production by *P. aeruginosa*. Biofilm staining with 1% crystal violet visualized under microscopic visualization. (b) Inhibition of biofilm production using the leaf extract from *X. americana* at the doses of 3.13 mg/mL (1/8 MIC) and 6.25 mg/mL (1/4 MIC). *n* = 4. ^∗^Significantly different at *p* < 0.05 from the control.

The opportunistic pathogenic bacterium, *P. aeruginosa*, commonly found in skin infections, has shown antimicrobial resistance due to its ability to form biofilms that embed the bacteria in a self-produced extracellular matrix. The biofilm is difficult to eradicate with antibiotic treatment as a limited number of antibiotics could diffuse easily through the matrix [51]. The MIC determination showed that the extract completely inhibited the growth of *P. aeruginosa* at 25 mg/mL. The doses 3.13 and 6.25 mg/mL, corresponding to 1/8 and 1/4 MIC, respectively, did not exert any significant inhibition of the tested strain; however, they inhibited the biofilm production of *P. aeruginosa* in a dose-dependent manner and were able to decrease biofilm amounts by 25 and 70%, respectively (Figure 3).

**Figure 4:**
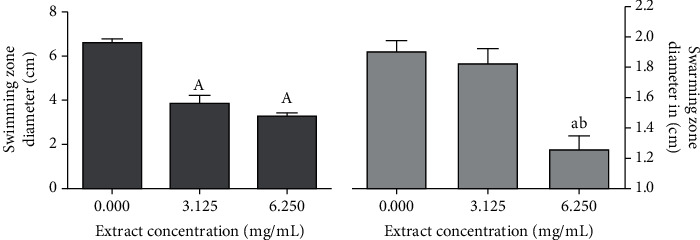
Effect of the leaf extract from *X. americana* on the swimming (a) and swarming mobility (b) of *P. aeruginosa* using the doses of 3.13 mg/mL (1/8 MIC) and 6.25 mg/mL (1/4 MIC). *n* = 4. ^a,b^Significantly different at *p* < 0.05 from the control and 3.13 mg/mL (1/8 MIC).

The mobility of *P. aeruginosa* on plates was impaired when the medium is amended with the extract in a dose-dependent manner (Figure 4). The extract reduced the swimming and swarming mobility by 41.35 and 4% at 3.23 mg/mL and by 50.37 and 34.21% at 6.25 mg/mL, respectively. Interestingly, the swimming mobility was significantly affected at 3.125 mg/mL but not the swarming mobility, which was significantly decreased only at 6.25 mg/mL (Figure 4). Similar activities were reported from polyphenol-rich extracts such as *Salix tetrasperma* (bark and flowers) [24].

## 4. Conclusions

In this study, a total of 23 mostly phenolic compounds were detected in a methanol leaf extract of *X. a.* var. *caffra*. The leaf extract showed substantial antioxidant activity *in vitro* and *in vivo* using the *C. elegans* animal model. It also inhibited four crucial enzymes of skin aging (collagenase, elastase, hyaluronidase, and tyrosinase) and the biofilm formation of *P. aeruginosa*. To sum up, the leaf extract exhibits interesting dermato-cosmeceutical properties that might be attributed to its antioxidant and antibacterial activities. Current findings could provide scientific evidence for possible utilization of the *X. a.* var. *caffra* leaf extract and its compounds as an antiaging agent.

## Figures and Tables

**Table 1:** Annotated secondary metabolites by HPLC-MS/MS analyses of the methanol extract of leaves from *X. americana* var. *caffra.*

No.	Proposed compounds	*t*_R_ (min.)	[M-H]^−^ (*m*/*z*)	MS/MS fragments
1	Quinic acid	1.41	191	
2	Coumaroyl-*O*-galloyl glucose	3.12	477	169, 313
3	Coumaroyl-*O*-galloyl glucose	3.38	477	169, 313
4	(epi)-Catechin-(epi)-catechin-(epi)-catechin	3.8	865	289, 577, 695
5	(epi)-Catechin carboxylic ester	4.38	333	245, 289, 315
6	(epi)-Catechin-(epi)-catechin	8.12	577	289, 407, 559
7	Procyanidin B1	9.18	577	289, 407, 559
8	Epicatechin	10.09	289	179, 205, 245
9	(epi)-Catechin-(epi)-catechin-(epi)-catechin	10.63	865	287, 577, 695
10	Catechin	14.42	289	179, 205, 245
11	Pyrogallol-*O*-methylgalloyl glucose	14.86	453	169, 313
12	Procyanidin dimer monogallate	15.61	729	289, 425, 577
13	(epi)-Catechin-(epi)-catechin	17.96	577	289, 407, 559
14	Quercetin galloyl-hexoside	20.46	615	179, 301, 463
15	Rutin	21.06	609	179, 301, 463
16	Isoquercetin	22.05	463	151, 179, 301
17	Avicularin	24.03	433	151, 179, 301
18	Kaempferol 3-*O*-glucoside	25.46	447	285
19	Kaempferol 3-neohesperidoside	26.13	593	285
20	Quercetin pentoside	26.5	433	151, 179, 301
21	Quercetin rhamnoside	27.47	447	151, 179, 301
22	Kaempferol 3-*O*-arabinoside	28.77	417	151, 179, 285
23	Kaempferol pentoside	31.46	417	151, 179, 285

**Table 2:** Antioxidant activities of the leaf methanol extract of *X. a. caffra.*

Sample	DPPH (EC_50_, *μ*g/mL)	FRAP (mM FeSO_4_/mg extract)
Leaf extract	5 ± 0.15	18.32 ± 0.32
Ascorbic acid (reference)	2.92 ± 0.29	
Quercetin (reference)		24.04 ± 1.23

**Table 3:** Enzymatic activities of the leaf methanol extract of *X. a. caffra.*

Enzyme	Elastase	Hyaluronidase	Tyrosinase	Collagenase
	IC_50_ (*μ*g/mL, mean ± SD)			
Extract	24.05 ± 2.53	13.66 ± 1.18	32.06 ± 1.18	39.55 ± 5.5
Kojic acid	21.60 ± 0.9	14.46 ± 0.6	9.00 ± 0.9	—
Quinic acid	143.75 ± 26.5	146.77 ± 11.3	61.91 ± 5.2	52.78 ± 1.8
Rutin	121.49 ± 22.8	57.39 ± 8.91	44.15 ± 5.25	63.46 ± 3.4
Catechin	15.00 ± 5.9	22.99 ± 0.2	22.54 ± 4.61	29.33 ± 2.4
Epicatechin	42.17 ± 5.7	53.39 ± 10.7	62.55 ± 2.3	65.96 ± 7.8

## Data Availability

All data are included in the manuscript.
